# Identification of regional overdistension, recruitment and cyclic alveolar collapse with electrical impedance tomography in an experimental ARDS model

**DOI:** 10.1186/s13054-016-1300-y

**Published:** 2016-05-03

**Authors:** Songqiao Liu, Li Tan, Knut Möller, Inez Frerichs, Tao Yu, Ling Liu, Yingzi Huang, Fengmei Guo, Jingyuan Xu, Yi Yang, Haibo Qiu, Zhanqi Zhao

**Affiliations:** Department of Critical Care Medicine, Zhongda Hospital, School of Medicine, Southeast University, Nanjing, Jiangsu Province 210009 China; Institute of Technical Medicine, Furtwangen University, Jakob-Kienzle Straße 17, 78054 VS-Schwenningen, Germany; Department of Anesthesiology and Intensive Care Medicine, University Medical Center of Schleswig-Holstein Campus Kiel, Arnold-Heller-Straße 3, 24105 Kiel, Germany

**Keywords:** Mechanical ventilation, Acute respiratory distress syndrome, Electrical impedance tomography, Recruitment/derecruitment, Overinflated

## Abstract

**Background:**

Information on regional ventilation distribution in mechanically ventilated patients is important to develop lung protective ventilation strategies. In the present prospective animal study, we introduce an electrical impedance tomography (EIT)-based method to classify lungs into normally ventilated, overinflated, tidally recruited/derecruited and recruited regions.

**Methods:**

Acute respiratory distress syndrome (ARDS) was introduced with repeated bronchoalveolar lavage in ten healthy male pigs until the ratio of arterial partial pressure of oxygen and fraction of inspired oxygen (PaO_2_/FiO_2_) decreased to less than 100 mmHg and remained stable for 30 minutes. Stepwise positive end-expiratory pressure (PEEP) increments were performed from 0 cmH_2_O to 30 cmH_2_O with 3 cmH_2_O increase every 5 minutes. Respiratory system compliance (Crs), blood gases and hemodynamics were measured at the same time. Lung regions at end-expiration and during tidal breathing were identified in EIT images.

**Results:**

Overinflated regions contain air at end-expiration but they are not or are only minimally ventilated. Recruited regions compared to reference PEEP level contain air at end-expiration of arbitrary PEEP level but not at that of reference PEEP level. Tidally recruited/derecruited regions are not represented in lung regions at end-expiration but are ventilated during tidal breathing. The results coincided with measurements of blood gases. The coefficient for correlation between the number of recruited pixels and PaO_2_/FiO_2_ was 0.89 ± 0.12 (*p* = 0.02).

**Conclusion:**

The proposed novel EIT-based method provides information on overinflation, recruitment and cyclic alveolar collapse at the bedside, which may improve the ventilation strategies used.

## Background

Acute respiratory distress syndrome (ARDS) is a life-threatening condition that requires mechanical ventilation in an intensive care unit. Despite all advanced management strategies, such as lung-protective ventilation, the mortality rate in severe ARDS is still very high [1]. Improper ventilator settings may lead to high transpulmonary pressures, alveolar overdistention, cyclic recruitment/derecruitment, and biotrauma, which induce or aggravate lung injury [2]. Lung-protective ventilation demands low tidal volume and appropriate airway pressure to open the atelectatic lung regions without overinflating other lung regions. Information on regional ventilation distribution in mechanically ventilated patients is important in the development of such strategies,. However, hardly any established clinical methods can fulfil this task. Computed tomography (CT) has high spatial resolution and is considered the gold standard for assessment of aeration in injured lungs [3, 4]. CT is not suitable for bedside assessment of dynamic information of respiration, such as tidal recruitment, not only because of the radiation load but also due to its relatively low temporal resolution.

Electrical impedance tomography (EIT) is the first bedside imaging technique that enables monitoring of air distribution in the lungs [5]. The basic idea of EIT is that changes in regional air content modify the electrical impedance of lung tissue [6]. Small alternating electrical currents are applied at the surface of the chest wall during the measurement and the resultant potential differences are recorded. The distribution of electrical impedance within the thorax is then calculated. The reliability of EIT has been confirmed by comparison with various conventional methods, such as CT [7, 8], single-photon-emission computed tomography [9], positron emission tomography [10], and pneumotachography [11].

In current EIT techniques, only relative impedance waveforms are reconstructed to minimize the influence of electrode positions, thorax shape, etc., and hence, no absolute volume is measured. In the case of overdistension and atelectasis, the relative change in impedance, which occurs during tidal breathing in babies with ARDS, is easy to determine using EIT. In the present study, we introduce a method to trace the lung regions where overinflation, recruitment and cyclic alveolar collapse have occurred.

## Methods

The study was approved by the Science and Technological Committee and the Animal Use and Care Committee of the Southeast University School of Medicine, Nanjing, China. All experiments were performed according to the Guidance for the Care and Use of Laboratory Animals [12].

### Animal preparation

A total of 10 healthy male pigs (body weight 51.2 ± 1.9 kg, mean ± SD) were included. Pigs were anesthetized with an intramuscular injection of ketamine hydrochloride (3 mg/kg), atropine (2 mg/kg) and fentanyl citrate (2 mg/kg), followed by a continuous intravenous infusion of propofol (1–2 mg/kg/h), fentanyl citrate (0.5–1.0 μg/kg/h), and midazolam (0.1 mg/kg/h), and atracurium (0.4 mg/kg/h). The animals were placed in the supine position on a thermo-controlled operation table to maintain body temperature at about 37.5 °C: 5 ml/kg of 0.9 % saline solution was administered during the anesthesia. After tracheostomy, the animals were mechanically ventilated (Servo-i ventilator, Solna, Sweden.) in a volume-controlled mode at the following settings. Respiratory frequency was set at 30 breaths per minute with an inspiration-to-expiration time ratio (I:E) of 1:2 and a positive end-expiratory pressure (PEEP) of 5 cm H_2_O. The fraction of inspiration of O_2_ (FiO_2_) and tidal volume (V_T_) were 0.4 and 6 ml/kg, respectively. A Swan–Ganz catheter (Arrow International, Reading, PA, USA) was inserted through the internal jugular vein to measure central venous pressure (CVP) and pulmonary arterial wedge pressure (PAWP). A thermistor-tipped PiCCO catheter (Pulsion Medical System, Munich, Germany) was advanced through the right femoral artery to monitor the mean arterial pressure (MAP). Additionally, arterial blood samples were collected from a PiCCO catheter. A continuous infusion of a 5 ml/kg/h balanced electrolyte solution was administered during the experiment, and MAP was maintained above 60 mmHg with rapid infusions of 0.9 % saline solution at up to 20 ml/kg, if required.

### Experiment protocol

After the initial preparation, the pigs were stabilized for 30 minutes and baseline measurements (T_Baseline_) were taken. ARDS was then induced by bilateral lung lavage with 30 ml/kg of isotonic saline (38 °C). The saline was infused through a funnel while the chest was gently massaged. After the maneuver, the excessive fluid was allowed to drain by gravity and was removed by negative pressure suction through the proximal portion of the endotracheal tube. The alveolar lavages were repeated once every 10 minutes until the PaO_2_/FiO_2_ ratio decreased to less than 100 mmHg and remained stable for 30 minutes (T_ARDS_) with an increase in FiO_2_ requirements to 1.0.

Stepwise PEEP increments were performed from 0 cmH_2_O to 30 cmH_2_O with 3 cmH_2_O increase every 5 minutes. Respiratory system compliance (Crs), and hemodynamic and gas exchange indices were measured and recorded at the following time periods: T_Baseline_, T_ARDS_, and at every pressure level during the incremental PEEP maneuver. CVP, PAWP, and MAP were monitored, using calibrated pressure transducers. All blood gas measurements were performed using an automated blood gas analyzer (Nova M; Nova Biomedical, Waltham, MA, USA).

### EIT measurements and analysis

EIT measurements were performed during the whole procedure described above (PulmoVista 500, Dräger Medical, Lübeck, Germany). An EIT electrode belt with 16 electrodes was placed around the thorax 5 cm above the xyphoid level and one reference electrocardiogram (ECG) electrode was placed at the abdomen. The frequency of injected alternating current was selected automatically according to the noise spectrum. The images were continuously recorded and reconstructed at 40 Hz. The baseline frames for reconstruction were selected as the minimum of the respective EIT data.

**Fig. 1:**
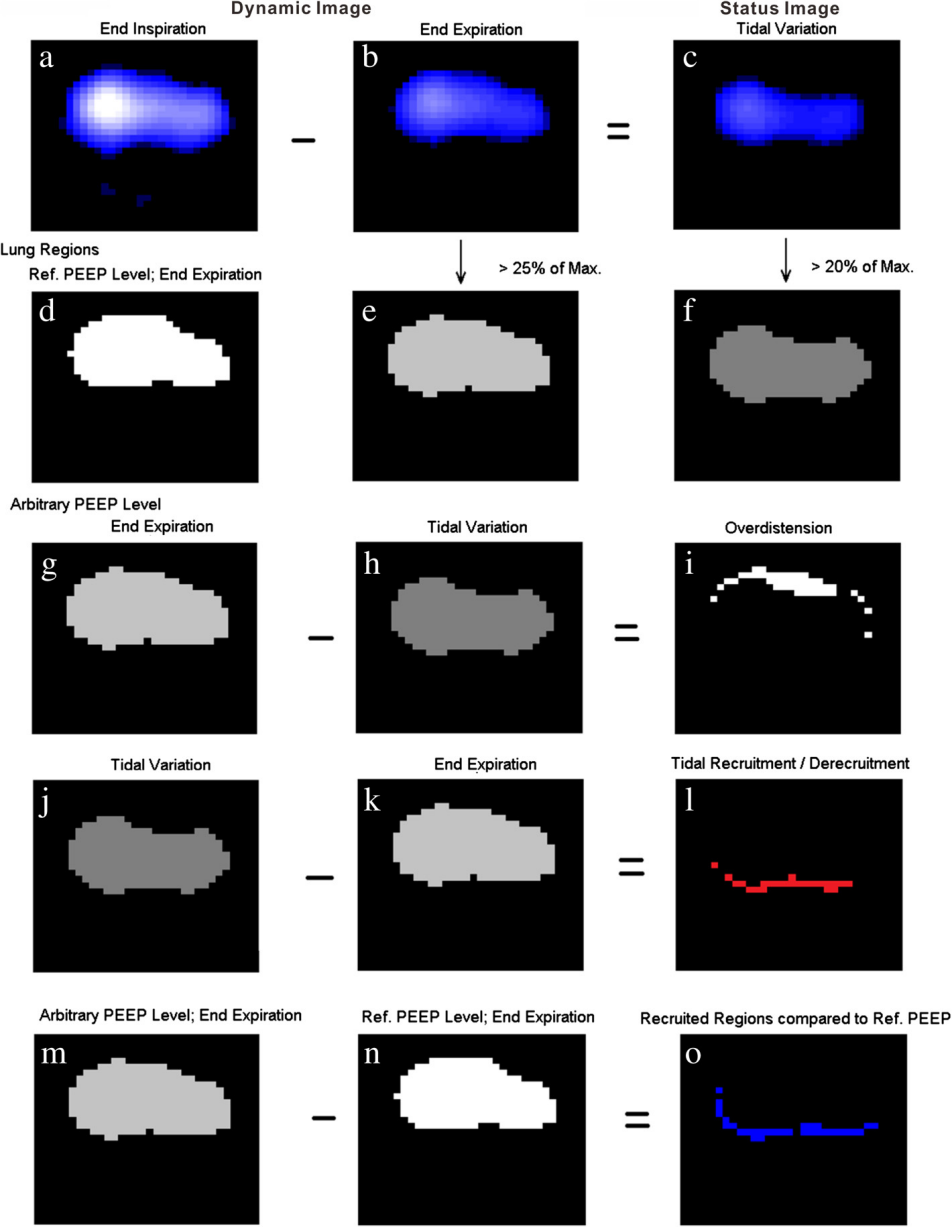
Illustration of the proposed analysis method. *First row* dynamic images at end-inspiration (**a**) and end-expiration (**b**) are identified (averaging of five corresponding images is used to minimize noise if necessary). Status image (**c**) of tidal variation equals pixel impedance values at end-inspiration minus that at end-expiration. *Second row* lung regions containing air are identified at reference positive end-expiratory pressure (*PEEP*) level and arbitrary PEEP level (**d**,* white regions* end-expiration at reference PEEP; **e**, *light gray* end-expiration at arbitrary PEEP; **f**, *gray* lung regions for tidal breathing). *Third row* overdistended regions (**i**,* white regions*) are defined as regions that contain air at end-expiration but no air comes in or out during tidal breathing. *Fourth row* regions subjected to repeated alveolar collapse and reopening (**l**,* red regions*) are defined as regions that are not in the lung regions at end-expiration but are ventilated during tidal breathing. *Fifth row* recruited regions compared to reference PEEP level (**o**,* blue regions*) are defined as regions that contain air at end-expiration of the arbitrary PEEP level but not at that of the reference PEEP level. For the combination of the three types of regions please refer to, e.g., Fig. 2, PEEP = 9 cmH_2_O

The method is schematically described in Fig. 1 using example data obtained in one of the animals. The analysis was performed as follows: five consecutive breaths at the end of each of the 10 studied PEEP steps were selected and EIT images at end-inspiration and end-expiration were identified. The five end-inspiration and the five end-expiration images were then averaged to minimize the influence of noise. An image was then calculated by subtracting the end-expiration from the end-inspiration image, and this is referred to as the tidal image (Fig. 1, first row). All these three types of EIT images consisted of 1024 image pixels.

In the next step, lung regions were identified (Fig. 1, second row). At end-expiration they included all pixels with values higher than 25 % of the maximum pixel value in the image. Lung regions for tidal breathing included all pixels in the tidal image that were higher than 20 % of the maximum value in the image. Regions were considered to be overinflated, if they belonged to those image pixels that were selected in lung regions at end-expiration but at the same time not ventilated during tidal ventilation and therefore not identified in the tidal image-based regions (Fig. 1, third row). Regions were considered to undergo cyclic alveolar collapse and reopening if they were ventilated during tidal breathing but collapsed at end-expiration (Fig. 1, fourth row). Finally, regions were considered to be recruited compared to the reference PEEP step, if they were included in the lung regions at end-expiration at the current PEEP step but not at the reference PEEP (Fig. 1, fifth row). A reference PEEP step was used exclusively for calculation of recruited regions. Apparently, if a high PEEP level or a PEEP after the recruitment maneuver was selected as reference PEEP, on comparison there were fewer recruited regions.

As the EIT images were reconstructed against the baseline frame with the lowest impedance values during zero PEEP, the amplitude of noise at a PEEP level of 3 cmH_2_O may have the same level as impedance values in the images of end-expirations. Therefore, we considered the reference PEEP to be at 6 cmH_2_O and calculated the regions exhibiting overinflation, cyclic collapse/reopening and recruitment at PEEP in the range of 9 to 30 cmH_2_O.

Data were processed with MATLAB (Ver. 7.2, MathWorks Inc., Natick, MA, USA). The absolute number of pixels of the different conditions were presented as the percentage of the total number of lung pixels. Lung regions at end-expiration in the highest PEEP level was used to calculate the total number of lung pixels. The Lilliefors test was used to evaluate the distribution of all data. For data that were not normally distributed, results were expressed as median and interquartile range. For normally distributed data, results were expressed as mean and standard deviation. Pearson’s linear correlation was calculated to assess the correlation between the number of recruited pixels and PaO_2_/FiO_2_. A *p* value <0.05 was considered statistically significant.

## Results

ARDS was successfully induced by repeated bronchoalveolar lavage in all 10 pigs. MAP decreased during PEEP increments; heart rate, CVP and PAWP increased along with PEEP, as shown in Table 1. In addition, plateau pressure (Pplat) and tidal volume (VT) are also summarized in Table 1.

**Table 1:** Hemodynamic and respiratory mechanism parameters

			PEEP (cmH_2_O)										
	T_Baseline_	T_ARDS_	0	3	6	9	12	15	18	21	24	27	30
HR (BPM)	66.5 ± 10.8	78.3 ± 15.6	84.2 ± 22.4	83.0 ± 20.34	86.1 ± 28.5	87.3 ± 34.7	91.4 ± 35.6	91.7 ± 33.8	96.4 ± 25.9	99.6 ± 27.2	105.3 ± 32.7	111.6 ± 39.0	121.6 ± 43.5
MAP (mmHg)	119.1 ± 12.9	129.5 ± 17.0	111.5 ± 29.3	109.9 ± 29.38	110.3 ± 24.1	111.5 ± 23.0	109.1 ± 23.0	107.5 ± 22.5	101.9 ± 21.2	87.8 ± 24.8	84.5 ± 27.1	76.3 ± 27.8	81.0 ± 30.5
CVP (mmHg)	5.3 ± 2.6	7.8 ± 3.0	7.5 ± 3.1	7.6 ± 3.21	7.6 ± 2.7	7.9 ± 2.4	8.4 ± 2.3	9.1 ± 2.5	10.3 ± 2.8	10.8 ± 3.1	11.9 ± 3.2	13.3 ± 3.8	12.4 ± 3.5
PAWP (mmHg)	7.4 ± 3.6	10.8 ± 3.8	8.7 ± 3.2	9.4 ± 3.1	9.9 ± 3.3	10.1 ± 3.3	11.8 ± 3.5	12.0 ± 2.9	12.9 ± 2.6	14.1 ± 3.1	15.7 ± 3.3	17.3 ± 4.1	18.4 ± 4.1
Pplat (cmH2O)	4.5 ± 1.1	20.3 ± 3.4	17.3 ± 3.6	18.0 ± 4.4	19.1 ± 4.5	19.3 ± 4.9	23.4 ± 5.8	25.7 ± 5.5	29.3 ± 2.9	31.6 ± 3.2	36.3 ± 3.7	40.6 ± 3.0	44.8 ± 4.6
Vt (ml/kg)	6.2 ± 0.9	6.0 ± 0.6	6.0 ± 0.3	6.1 ± 0.2	6.3 ± 0.1	6.2 ± 0.1	6.1 ± 0.1	6.1 ± 0.5	6.0 ± 0.2	6.1 ± 0.2	6.0 ± 0.1	6.3 ± 0.4	6.0 ± 0.5

Data are expressed as mean ± SD. *PEEP* positive end-expiratory pressure, *T* time point, *ARDS* acute respiratory distress syndrome, *HR* heart rate, *MAP* mean arterial pressure, *BPM* beats per minute, *CVP* central venous pressure, *PAWP* pulmonary arterial wedge pressure, *Pplat* plateau pressure, *VT* tidal volume

**Fig. 2:**
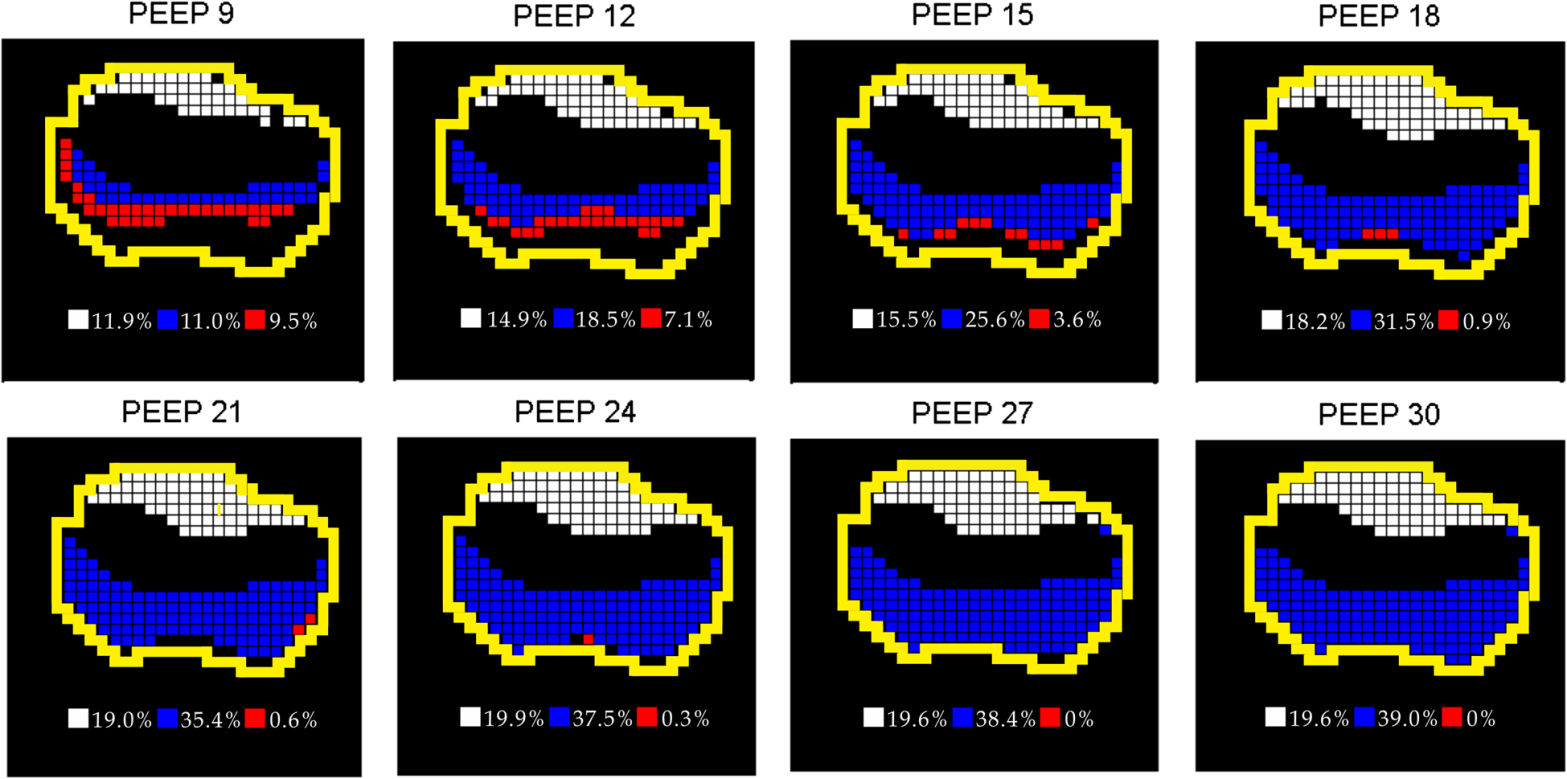
Regions that were identified as overdistended (*white*), recruited (*blue*) and tidally recruited/derecruited (*red*) during the incremental positive end-expiratory pressure (*PEEP*) trial in one pig. PEEP increased from 9 to 30 cmH_2_O. Recruited regions were calculated compared to a PEEP of 6 cmH_2_O. Lung regions are outlined in *yellow*. Percentage values at the bottom of each sub-figure were calculated by dividing theabsolute number of pixels of the different conditions by the total number of lung pixels

**Fig. 3:**
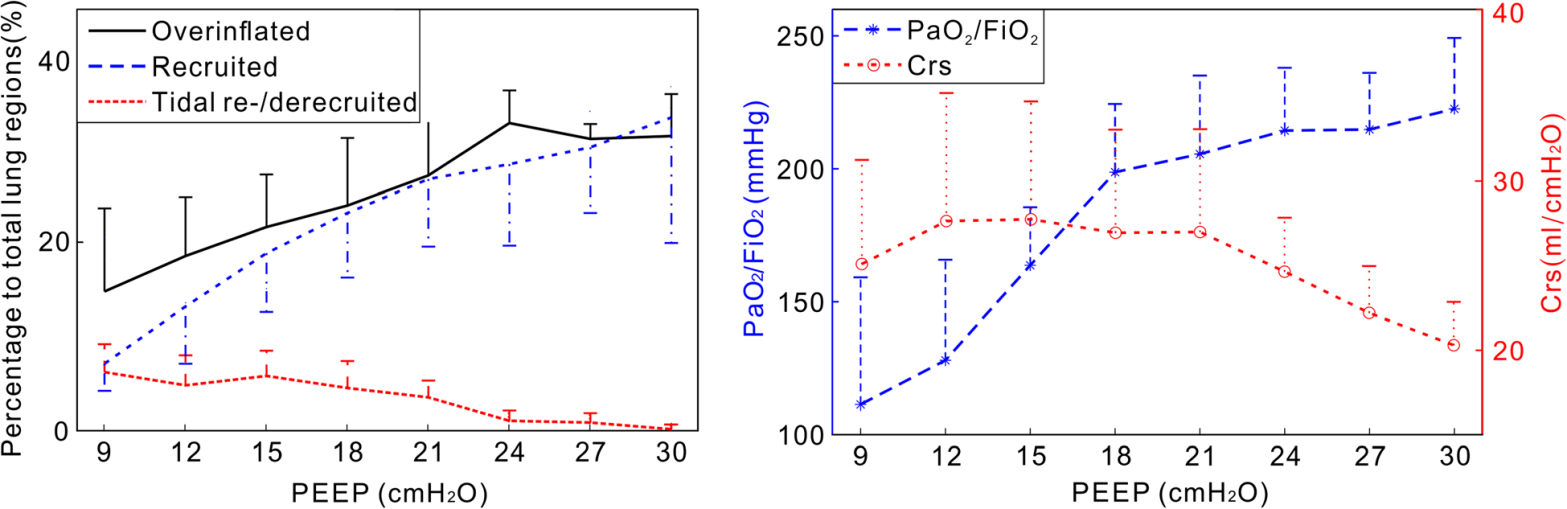
Summary of results obtained in all 10 pigs during the incremental positive end-expiratory pressure (*PEEP*) trial. *Left* number of pixels in overinflated (*black solid line*), recruited (*blue broken line*) and tidally recruited/derecruited (*red broken line*) regions divided by total number of lung pixels in percentage (medians and half interquartile ranges). Recruited regions were calculated compared to a PEEP of 6 cmH_2_O. The partial pressure of O_2_ in arterial blood/fraction of inspired oxygen (*PaO*_*2*_ */FiO*_*2*_) ratio (*blue stars*) and respiratory system compliance (*Crs*, *red circles*) are presented as median and half-interquartile ranges

Figure 2 shows regions exhibiting overinflation, cyclic collapse/reopening and recruitment identified in one pig. The white, overinflated regions existed already at the PEEP of 9 cmH_2_O and increased together with PEEP. The red regions disappeared at PEEP levels higher than 18 cmH_2_O, which implied that lower PEEP levels might cause cyclic tidal collapse and reopening of alveoli. The rate of increase in recruited blue regions (compared to PEEP = 6 cmH_2_O) decreased with PEEP. Although the numbers of pixels of these three regions differed individually, the trends in their changes with PEEP were similar in all 10 pigs (Fig. 3, left). PaO_2_/FiO_2_ increased with PEEP increments, while respiratory system compliance reached its highest value at about 12 to 21 cmH_2_O (Fig. 3, right).

The coefficient for correlation between the number of recruited pixels and PaO_2_/FiO_2_ was 0.89 ± 0.12 (*p* = 0.02); between the number of overinflated pixels and PAWP it was 0.84 ± 0.16 (*p* < 0.05). There was no significant correlation between the number of overinflated pixels and Crs (*p* > 0.05).

## Discussion

In the present study, we introduced a method to classify EIT images into normally ventilated, overinflated, recruited, and tidally recruited/derecruited regions. We tested our novel method prospectively in 10 ARDS-model pigs. The number of recruited pixels coincided with measurements of blood gases. The method gives intuitive information on overdistension and recruitment for intensivists. To our knowledge, it is the first method that directly assesses tidal recruitment/derecruitment quantitatively at the bedside.

EIT possesses several advantages when compared to established imaging techniques. CT, as the current gold standard, exposes the patients to radiation, despite the doses being relatively low. Also, in clinical practice, CT pixels presenting densities smaller than –850 Hounsfield Unit (HU) are defined as representing overdistension. But in the present EIT data analysis, we defined regions as representing overdistension when they had high air levels on end-expiration but no air coming in or out during tidal breathing. Hence, our EIT analysis is, per se, closer to the definition of overdistension. In previous studies, we developed a so-called global inhomogeneity (GI) index to quantify the ventilation distribution [13, 14]. The degree of atelectasis and overdistension was represented by the GI index. However, calculation of the GI index requires identification of the lung regions, which is not always easy and exact withg different lung volumes [15]. Meier et al*.* [16] and Bikker et al*.* [17] used differences in images between consecutive PEEP steps to identify regional volume gain and loss. Although this information gave an intuitive impression of regions gaining or losing air, due to the nature of relative impedance, it was hard to judge the amount of gain or loss that was critical to the patient. The display of information depends also on the chosen color scale. Costa et al*.* proposed a PEEP titration method with regional compliance [18]. They defined atelectasis and overdistension based on regional compliance values after or before their best values. Comparison with CT showed good correlation between alveolar collapse and overestimation of overdistension. However, this method requires a full titration process, i.e., if the number of PEEP steps is too small to produce variable regional compliance changes, neither atelectasis nor overdistension can be identified. Further, these methods used only the information on tidal variation to assess atelectasis and overdistension. Information at end-expiration was ignored.

Dargaville et al*.* plotted regional pressure-volume loops with end-expiratory lung impedance values at each step of an incremental and decremental PEEP trial [19]. Zick et al*.* calculated the center of ventilation and regional Crs at various ventral-dorsal layers [20]. These kinds of assessment of regional Crs also allowed identification of overinflation and recruitment. However, all these studies required PEEP changes or tidal volume changes to introduce changes in tidal variation. Our novel method identifies three different types of region. Only the calculation of recruited regions requires reference PEEP for comparison. Calculation of the other two types of region compares information on tidal variation with end-expiration at the same PEEP step, and therefore can be applied at a single PEEP level. In the present study, we started evaluation from a PEEP of 6 cmH_2_O to avoid the influence of noise. In practice, only a baseline with low impedance is required for image reconstruction. Muders et al*.* proposed a regional ventilation delay index (RVD) to estimate the amount of tidal recruitment [21]. However, this method required a special maneuver (low-flow maneuver) to prolong the inspiration phase, which may not correctly reflect normal tidal recruitment. In normal breathings where inspiration time is shorter, RVD becomes unstable.

To evaluate the proposed method, we performed an incremental instead of a decremental PEEP trial on pigs undergoing lavage. Due to viscoelasticity, more distinct changes may be observed in three types of region before a recruitment maneuver is performed. A fairly high PEEP level was used, which may drive the diaphragm towards the abdomen and change the lung regions in the EIT measuring plane. We placed the EIT electrodes around the thorax 5 cm above the xyphoid level to minimize this influence.

We observed that overdistended regions decreased slightly at the highest PEEP levels in 3 out of 10 pigs. We suspected that part of the overdistended regions were ventilated again at the highest PEEP, as most of the lung regions were overdistended, but air delivered from the ventilator had to be distributed somewhere in the lungs. We consider a decrease in the overdistended regions at the highest PEEP levels as a very dangerous sign, which may induce pneumothorax. Based on the results of the proposed method, we do not recommend an optimal PEEP. Instead, this method provides extra information about overdistension and tidal recruitment/derecruitment. Together with other information, such as arterial oxygenation and disease status, the ventilation strategies can be improved accordingly with more confidence. A previous study showed that there is a strong and inverse relationship between arterial oxygenation and the percentage of collapsed lung tissue observed on CT [22]. Our results also show that there was a significant correlation between the number of recruited pixels and PaO_2_/FiO_2_. Although oxygenation is a global parameter, it is indirectly correlated with the local ventilation distribution.

Our study has several limitations. One is that there was no reference method. Studies show that EIT assesses the ventilation distribution during mechanical ventilation as reliably as CT [8, 23]. In fact, no established techniques provide similar dynamic information at the bedside except for EIT. CT is considered the gold standard for assessment of aeration and could be used at both end-inspiration and end-expiration to validate the EIT method as presented. Using CT we could use the absolute densities in HU to define overdistension. However, the differences in lung size and shape on end-inspiration and end-expiration make it hard to dynamically compare the sizes of the overdistended and collapsed regions. We still could not confirm the regions of tidal recruitment/derecruitment by using CT. Another limitation of the method is that threshold values have to be preset for identification of the lung regions. Images at end-expiration are dynamic, containing a higher level of noise compared to tidal images, which are status images caused by inspiration and expiration. Therefore, in the present study, the threshold of lung regions for end-expiration is 5 % higher than that for tidal images.

Pulletz et al*.* had studied the influence of threshold values on the image evaluation and they found that threshold values between 20 and 35 % might be suitable [24]. However, we have little information about the optimal threshold for the proposed method. We have tested other threshold combinations and found that changes in the threshold would alter the number of pixels in the regions (increasing the threshold values decreases the number of pixels for overdistension and recruitment), but fortunately the trends were independent from threshold selection (overdistended and recruited regions increase with PEEP and regions of tidal recruitment/derecruitment decrease with PEEP), which are important for decision making. The relatively high amount of overdistension observed at low PEEP levels might be due to difficulty in measuring overdistension with EIT, and thus, has to be interpreted with caution. We presented the degree of overdistension and recruitment relative to the total number of lung pixels to minimize the dependence on the lung region definition threshold. Last, even though the lung lavage model is a widely used lung injury model, it is not an ideal ARDS model because it responds very well to PEEP recruitment. However, on the other hand, the efficiency of our method could ideally be demonstrated through this model.

## Conclusions

The proposed novel EIT-based method provides information on overdistension, recruitment and cyclic alveolar collapse at the bedside, which may improve the titration of optimal ventilator settings.

## Key messages

- The ventilation distributions at end-inspiration and end-expiration measured with EIT provide extra dynamic information on the lungs in ARDS.
- The novel EIT-based method provides information on overdistension, recruitment and cyclic alveolar collapse, at the bedside.
- Thorax EIT measurement is robust and has the potential to improve the titration of optimal ventilator settings.

## Abbreviations

ARDS: acute respiratory distress syndrome
Crs: respiratory system compliance
CT: computed tomography
CVP: central venous pressure
EIT: electrical impedance tomography
FiO_2_: fraction of inspired oxygen
GI: global inhomogeneity
HU: Hounsfield units
MAP: mean arterial pressure
PaCO_2_: partial pressure of CO_2_ in arterial blood
PaO_2_: partial pressure of O_2_ in arterial blood, PAWP, pulmonary arterial wedge pressure
PEEP: positive end-expiratory pressure
Pplat: plateau pressure
RVD: regional ventilation delay index
VT: tidal volume
